# Synthesis of Hydrophilic Poly(butylene succinate-butylene dilinoleate) (PBS-DLS) Copolymers Containing Poly(Ethylene Glycol) (PEG) of Variable Molecular Weights

**DOI:** 10.3390/polym13183177

**Published:** 2021-09-18

**Authors:** Moein Zarei, Miroslawa El Fray

**Affiliations:** Department of Polymer and Biomaterials Science, Faculty of Chemical Technology and Engineering, West Pomeranian University of Technology inSzczecin, Al. Piastow 45, 71-311 Szczecin, Poland; zm47832@zut.edu.pl

**Keywords:** poly(butylene succinate), poly(ethylene glycol), polycondensation, wettability

## Abstract

Polymeric materials have numerous applications from the industrial to medical fields because of their vast controllable properties. In this study, we aimed to synthesize series of poly(butylene succinate-dilinoleic succinate-ethylene glycol succinate) (PBS-DLS-PEG) copolymers, by two-step polycondensation using a heterogeneous catalyst and a two-step process. PEG of different molecular weights, namely, 1000 g/mol and 6000 g/mol, was used in order to study its effect on the surface and thermal properties. The amount of the PBS hard segment in all copolymers was fixed at 70 wt%, while different ratios between the soft segments (DLS and PEG) were applied. The chemical structure of PBS-DLS-PEG was evaluated using Fourier transform infrared spectroscopy and nuclear magnetic resonance spectroscopy. Gel permeation chromatography was used to determine the molecular weight and dispersity index. The results of structural analysis indicate the incorporation of PEG in the macrochain. The physical and thermal properties of the newly synthesized copolymers were also evaluated using water contact angle measurements, differential scanning calorimetry and dynamic thermomechanical analysis. It was found that increasing the amount of PEG of a higher molecular weight increased the surface wettability of the new materials while maintaining their thermal properties. Importantly, the two-step melt polycondensation allowed a direct fabrication of a polymeric filament with a well-controlled diameter directly from the reactor. The obtained results clearly show that the use of two-step polycondensation in the melt allows obtaining novel PBS-DLS-PEG copolymers and creates new opportunities for the controlled processing of these hydrophilic and thermally stable copolymers for 3D printing technology, which is increasingly used in medical techniques.

## 1. Introduction

Polymers, which are derived from renewable resources, are becoming increasingly more important as they are one of the key approaches in solving environmental problems such as increasing pollution and energy shortages caused by petroleum consumption [1,2]. Another great advantage is their use in medical applications, including tissue engineering in soft and hard tissue repair, drug delivery systems and resorbable sutures [3,4,5,6]. While the majority of research in recent decades has been focused on poly(lactic acid)/polylactide (PLA) and its copolymers, increasing interest is being directed towards another aliphatic polyester, poly(butylene succinate) (PBS), characterized by interesting physical and biological properties [7]. First of all, it is derived from succinic acid (SA), either derived from biomass or as endogen metabolite monomer, imparting biodegradability and high biocompatibility to PBS. Secondly, its inherent biodegradation, providing nontoxic degradation products, excellent mechanical properties and thermoplastic processability and in vitro biocompatibility in the presence of different animal and human cell lines have made PBS suitable for various medical applications [8]. Porous structures prepared from PBS by solvent casting have been reported as promising scaffolds for bone cell growth, as they have been demonstrated to support the attachment of mouse calvaria-derived pre-osteoblastic cells on their surface [9]. In another study, an excellent cell response to PBS was reported in the presence of human mesenchymal stem cells, making this polymer an excellent candidate for tissue engineering applications [10]. A notable feature of PBS is the ability to be easily tailored by copolymerization with other comonomers for adjusting its properties, especially for imparting elasticity or hydrophilicity (the PBS homopolymer is a relatively stiff and hydrophobic material).

Recently, new biobased copolyesters have been synthesized using PBS constituting the hard segments in multiblock copolymers, while dimer linoleic acid (DLA) or dimer linoleic diol (DLA-OH) units were used as the soft (flexible) segment component [11]. These copolymers have demonstrated excellent flexibility (up to 600% strain at break) with controllable biodegradation and high biocompatibility [12]. By changing the PBS-to-DLA/DLA-OH (hard-to-soft segment) ratio, poly(butylene succinate-co-butylene dilinoleate) (PBS-DLA) or poly(butylene succinate-dilinoleic succinate) (PBS-DLS) copolymers, covering a wide range of mechanical properties (from stiff to flexible materials) and degradation rates, can be synthesized.

The excellent properties of poly(ethylene glycol) (PEG) such as its hydrophilicity and biocompatibility have allowed this aliphatic polyether to be approved by the Food and Drug Administration (FDA) for various medical applications [13]. Being commercially available in different molecular weights, it offers an interesting possibility to use it in the synthesis of multiblock copolymers with butylene terephthalate and butylene succinate units [14], highly suitable for controlled release systems. Such copolymers have demonstrated high swelling in water with increasing PEG segment length and higher in vitro degradation as the molecular weight of 1000 g/mol was used in comparison to copolymers synthesized with shorter PEG segments. In another study, multiblock PBS copolymers containing a biocompatible PEG block as a switching segment and PBS as a crystallizable hard segment were also synthesized by one-step polycondensation from succinic acid, 1,4-butanediol and PEG diol [15]. The results revealed that all PBS-PEG copolymers showed an adjustable melting point (T_m_) due to the easily crystallizable PBS hard segments. It has also been reported that PBS-PEG showed microphase separation leading to a multiblock architecture. The results of the tensile test indicated that when both the soft and hard segments had sufficient crystallinity, the PBS-PEG copolymers exhibit excellent shape memory properties. The hydrophilicity of PBS-PEG copolymers has also been improved by the incorporation of the hydrophilic PEG. These biodegradable PBS-PEG multiblock copolymers with excellent shape memory properties showed great potential for application in medical devices [16,17].

Therefore, considering all the advantages offered by segmented copolymers, where flexibility and hydrophilicity can be tailored by proper selection of building blocks, the purpose of this research was to create, for the first time, new PBS-DLS-PEG copolymers by using a new heterogenous titanium dioxide/silicon dioxide coprecipitate catalyst (instead of the common, typically used tetrabutoxy titanate) and performing the synthesis in two separate processes: transesterification followed by polycondensation in the melt. The long-chain fatty acid content was set at 30 wt % to provide sufficient flexibility at a reasonable stiffness, while different molecular weights of PEG (1000 g/mol and 6000 g/mol) and different ratios were used to fine-tune the hydrophilicity and crystallinity of the new copolymers for further material processing. Considering an increasing interest in computer-aided manufacturing, the aim was also to fabricate a filament directly from the reactor as our future work will be focused on fused deposition modeling (FDM) of hierarchical structures with the use of these new copolymers. 

## 2. Materials and Methods

### 2.1. Materials

Dimethyl succinate (DMS, 98%, CAS: 106-65-0, molecular weight: 146.14 g/mol) and poly(ethylene glycol) (PEG, CAS: 25322-68-3, average molecular weight: 1000 g/mol and 6000 g/mol) were purchased from Sigma Aldrich (ul. Szelągowska 30, 61-626, Poznań, Poland). 1,4-Butanediol (BD), 99%, CAS: 110-63-4) was purchased from Alfa Aesar (ThermoFisher GmbH, Erlangen, Germany), Pripol 2033 high-purity dimer diol (DLA-OH) was purchased from Croda (Wadowicka 6, 30-415 Kraków, Poland) and the titanium dioxide/silica dioxide heterogenous catalyst (C-94) was purchased from Huntsman, Duisburg, Germany.

### 2.2. Synthesis of PBS-DLS-PEG

**Figure 1:**
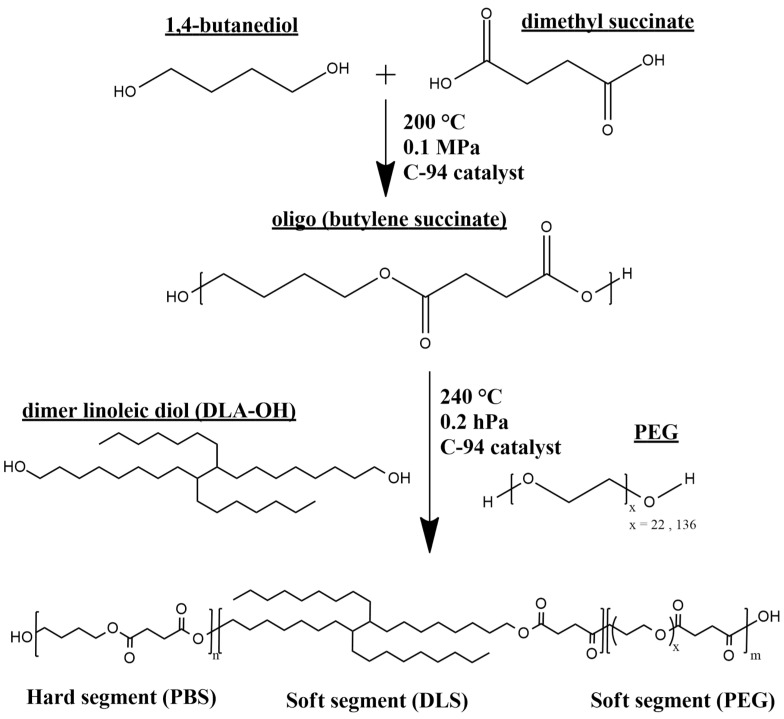
Schematic synthesis of PBS-DLS-PEG copolymers.

A series of multiblock copolymers containing butylene succinate (PBS) as hard segments, dilinoleic succinate (DLS) derived from dimer linoleic diol (DLA-OH) and poly(ethylene glycol) (PEG) with two different molecular weights (1000 g/mol and 6000 g/mol) in soft segments were synthesized by a two step-process, namely, transesterification and polycondensation in the melt. The synthesis scheme is presented in Figure 1. The weight ratio of the hard segments (PBS) was set at 70 wt %, while the content of soft segments was 30 wt %. In a three-component system, the ratio of DLS to PEG segments was set as 25:5 wt % and 20:10 wt %. PEG1000 g/mol and 6000 g/mol, designated as PEG_1000_ and PEG_6000_, were used for the synthesis. The transesterification stage was carried out in the presence of dimethyl succinate and 1,4-butanediol catalyzed by the titanium dioxide/silica dioxide heterogenous catalyst (C-94) at a temperature of 200 °C, until the theoretical amount of the condensate (methanol) was collected. After that, for the polycondensation step, DLA-OH and PEG were added to the reaction mixture, and then the pressure was decreased to 0.2–0.4 mbar, and the temperature was increased to 240 °C. Due to the excellent thermal stability of DLA-OH, the process was carried out without the usage of a thermal stabilizer. The progress of the polycondensation reaction was observed by the power consumption of the stirrer. After the reaction was complete (140 min), the formed copolymer was extracted from the reactor to the water bath in a form of solid filament for further examinations. Table 1 shows a summary of the copolymer compositions and conditions of the synthesis. 

### 2.3. Characterization of Chemical Structure

Proton and carbon nuclear magnetic resonance (^1^H NMR and ^13^C NMR), attenuated total reflectance (ATR) and Fourier transform infrared (FTIR) spectroscopies were employed to characterize the chemical structures of the new polymers. Using a Bruker Alpha spectrophotometer (Bruker Optik, Ettlingen, Germany) the IR spectra were collected for PEG, PBS-DLS and PBS-DLS-PEG copolymers. The ATR FT-IR spectra were recorded from 400 to 4000 cm^−1^ at a resolution of 2 cm^−1^ with 32 scans. A Bruker DPX 400 spectrometer (400 MHz) was used to record ^1^ H NMR spectra (128 scans, 1 s relaxation delay) and ^13^C NMR spectra (5120 scans, 1 s relaxation delay). The samples were dissolved in CDCl_3_ (40 mg/mL for ^1^H NMR and 80 mg/mL for ^13^C NMR), and tetramethylsilane (TMS) was employed as an internal reference for the reported chemical shifts. The calculation of the molecular weight and the experimental weight ratio of hard and soft segments was performed considering ^1^ H NMR data through Equation (1):

(1) $$DP_{h}=\frac{\frac{I_{H}}{n_{H}}}{\frac{I_{S}}{n_{S}}}$$

where *DP_h_* is the degree of polymerization of PBS hard segments, *I_H_* is the integral of the PBS hard segment, and *I_S_* is the integral of the DLS and PEG soft segments. The weight percentages of the hard segments (%*W_h_*) of the new copolyesters were also calculated from *DP_h_* using Equation (2):

(2) $$\%W_{h}=\frac{DP_{h}\cdot M_{h}}{\left(DP_{h}\cdot M_{h}\right)+M_{s}}$$

where *M_h_* is the molecular weight of the hard segment (172 g/mol), and *M_s_* is the molecular weight of the soft segment (624 g/mol for DLS, 1000 g/mol for PEG_1000_ and 6000 g/mol for PEG_6000_). 

### 2.4. Gel Permeation Chromatography (GPC)

The molecular weight of the obtained polymers was also determined using gel permeation chromatography (GPC). Measurements were performed on an HPLC Agilent 1200 series modular system with a refractive index detector (RID). The system was equipped with two PLgel 5 µm MIXED-C columns (300 × 7.5 mm) connected in series. Calibration was performed on 12 polystyrene standards with masses ranging from 474 to 1,800,000 g/mol. The measurements were performed at 35 °C. HPLC purity chloroform (CHCl_3_) with a flow rate of 0.7 mL/min was used as the mobile phase. Samples of 3 mg/mL concentration were filtered through a polytetrafluoroethylene (PTFE) membrane with a pore size of 0.2 µm before analysis. Data were recorded using “ChemStation for LC” and analyzed using “ChemStation GPC Data Analysis Software”.

### 2.5. Viscosity

The intrinsic viscosity (η) of the synthesized PBS-DLS-PEG was measured using an Ubbelohde viscometer (K = 0.00323) using chloroform as the solvent and immersing it in a water bath at 25.00 ± 0.1 °C. Polymers were dried for 48 h and dissolved in CHCl_3_ to obtain a concentration of 0.5 g/100 cm^3^, and the flow time of the solutions was recorded in 5 repetitions. The intrinsic viscosity values were calculated according to the Solomon–Ciuta equation [18].

### 2.6. Melt Flow Index (MFI)

The MFI of the new copolymers was measured at 120 °C with 2.16 kg of load according to ISO 1131-1991 (PN-93/C-89069) using a CAEST type CA-MAN-001. The MFI was calculated according to Equation (3):

(3) $$\mathrm{MFI}=\frac{600\times \;m}{t}$$

where 600 stands for the reference time (10 min = 600 s), m stands for the mass of the sample after the desired period (g), and *t* stands for the period of time (s).

### 2.7. Water Contact Angle

The water contact angle measurements were carried out to evaluate the effect of PEG on the surface properties of the synthesized copolymers. The water contact angle was measured by sessile drop shape analysis according to European Standard EN 828 using a goniometer Krüss DSA 100 Drop Shape Analyzer equipped with a camera and recording system. An amount of 2 µL ultra-pure deionized water drop was dripped on different areas of the sample’s films prepared by a hydraulic hot press (ReMi-Plast PH10T, Czerwonak, Poland). The contact angle was measured for a period from 0 to 60 s. Contact angle values for 15, 30 and 60 s were selected as the measurement points. The average angle was calculated from 5 measurements.

### 2.8. Thermal Properties

A differential scanning calorimeter, DSC Q2500 Discovery (TA Instruments Inc., New Castle, DE, USA), was used to evaluate the thermal properties of the new PBS-DLS-PEG copolymers during the heating–cooling–heating cycles. The samples of different groups were placed into the aluminum pans with the same weight (20 mg), and the measurements were carried out over the temperature range from −90 to 200 °C. The heating–cooling rate was 10 °C /min, and Trios software was used to analyze the obtained thermograms. The glass transition temperature (T_g_), crystallization temperature (T_c_) and melting temperature (T_m_) were determined. The degree of crystallinity (X_c,h_) of the PBS (hard segments) was determined by the measurement of the enthalpy of fusion and its normalization to the enthalpy of fusion of 100% crystalline PBS using the formula

X_c,h_ = ΔH_PBS_/(ΔH_PBS_^*^ × W_PBS_) × 100 (4)

where ΔH_PBS_ stands for the melting enthalpy of PBS in the polymer, ΔH_PBS_^*^ is the heat of fusion of PBS with 100% crystallinity at the equilibrium melting temperature (102 J/g), and W_PBS_ is the mass fraction of PBS. 

### 2.9. Dynamic Mechanical Thermal Properties

Dynamic mechanical thermal analysis (DMTA) was carried out with a DMA Q800 (TA Instruments, New Castle, Delaware, USA) using the RHIOS 4.4.4 software with a 1 Hz frequency over a temperature range of −100 to 150 °C, with a heating rate of 3 °C/min, using the rectangular center parts of the micro-dumbbell specimens prepared by a hydraulic hot press.

## 3. Results and Discussion

### 3.1. Chemical Structure

**Figure 2:**
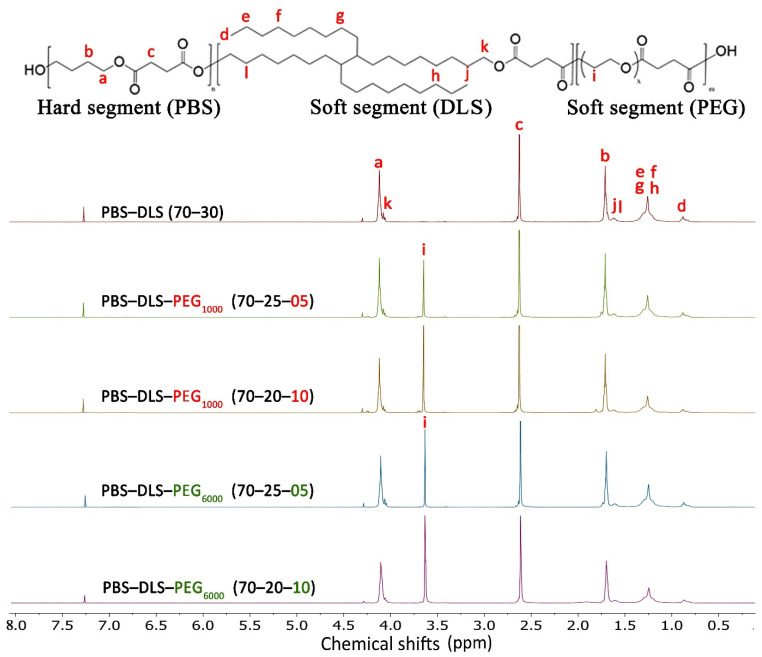
^1^H NMR spectra of synthesized PBS-DLS, PBS-DLS-PEG_1000_ and PBS-DLS-PEG_6000_ of different segmental compositions.

The series of new PBS-DLS-PEG copolymers were synthesized via a two-step transesterification and polycondensation process using a heterogenous titanium dioxide/silicon dioxide coprecipitate catalyst. Using this catalyst can bring some important advantages to the synthesis process over the organometallic tetrabutoxy titanate (TBT) catalyst that is typically used for PBS synthesis [19]. This solid powder heterogenous catalyst does not require distillation before use because of its hydrolytic stability, and compared to the hazardous TBT, it has less environmental, health and safety impacts [19]. The chemical structure of the newly synthesized polymers was assessed by ^1^H NMR and ^13^C NMR spectra. The signals that appeared in ^1^H NMR related to the copolymer series with PEG_1000_ and PEG_6000_ are presented in Figure 2. It can be seen that the main difference in the chemical structure after the addition of PEG is presented by the signals at δ^1^H = 3.68 ppm (i) which are assigned to the methylene units of PEG attached to the ether group [15]. By increasing the percentage of PEG from 5 to 10%, the intensity of these signals increases, thus confirming the incorporation of PEG into the polymer. The signal appearing at δ^1^H = 4.12 ppm (a) corresponds to the outer methylene protons of PBS, which also confirms the formation of a BD-DMS ester bond, and the signal at δ^1^H = 1.71 ppm (b) corresponds to the inner methylene protons of PBS. The ester bond formation between DLS-OH and DMS appeared at δ^1^H = 4.06–4.08 ppm (k). The signal at δ^1^H = 4.12 (a) also corresponds to the protons of PBS which were attached to the ester group. The methylene protons of DMS also appeared at δ^1^H = 2.63 (c). The results from ^1^H NMR analysis confirm the expected chemical structure of the newly synthesized PBS-DLS-PEG copolymers. 

**Figure 3:**
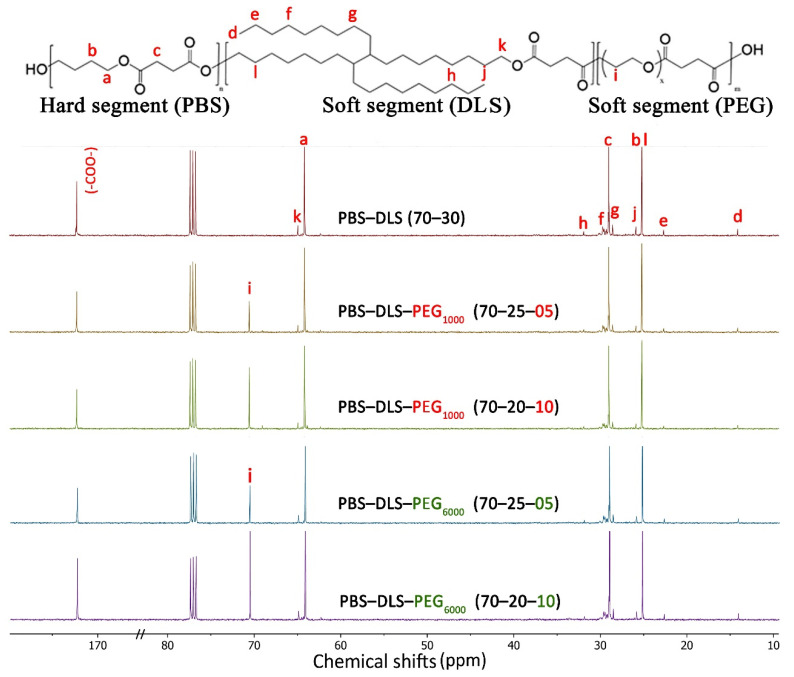
^13^C NMR spectra of synthesized PBS-DLS, PBS-DLS-PEG_1000_ and PBS-DLS-PEG_6000_ of different segmental compositions.

The analysis of ^13^C-NMR spectra (Figure 3) revealed the appearance of a signal at δ^13^C = 70.57 ppm (i) assigned to PEG. It can also be seen that for the higher-molecular weight PEG_6000_, the signals appeared at a higher intensity, indicating a higher amount of PEG in the copolymers. The DMS carbon atoms of the formed carboxyl groups (–COO–) attached to BD and attached to the DLA-OH sequence appeared at δ^13^C = 172.27 ppm and δ^13^C = 172.30 ppm, respectively. The signals that appeared at 64.17 ppm (a) and at 64.94 ppm (k) are responsible for the reaction between BD and DMS and the reaction between DLA–OH and DMS, respectively. The signals at 29.02 ppm (c) and at 25.21 ppm (b) are related to –CH_2_– from the DMS and BD units, respectively. The low-intensity signals at 25.6 ppm (j), 29.09 ppm (f) and 14.2 ppm (d) are also related to the DLS soft segment. 

### 3.2. Infrared Spectroscopy (ATR FT-IR)

**Figure 4:**
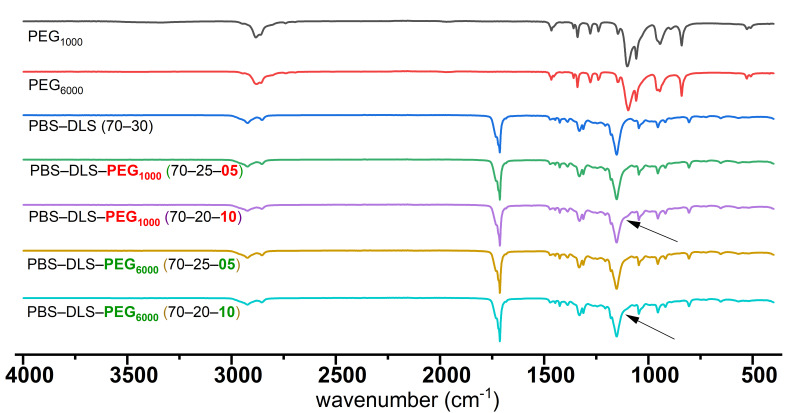
FTIR spectra of synthesized PBS-DLS, PBS-DLS-PEG_1000_ and PBS-DLS-PEG_6000_ of different segmental compositions (arrows indicate small band related to PEG).

The chemical structures of the synthesized PBS-DLS-PEG copolymers were also characterized by ATR FT-IR spectroscopy. Figure 4 represents the IR spectra for the polymer series containing PEG_1000_ and PEG_6000_. For the PBS-DLS copolymer, the absorption bands at 2920 cm^−1^ and 2855 cm^−1^ are assigned to the stretching vibration of the methylene groups (-CH_2_-) of the soft segments. The C=O carbonyl vibrations and ester C-O-C bond stretching appeared at around 1710 cm^−1^ and 1150 cm^−1^, respectively. After introducing PEG into the copolymers, the PBS-DLS-PEG (1000 and 6000) materials showed small bands at around 1070 cm^−1^ which further increased in intensity with a higher concentration of PEG (Figure 4; arrows), and this band is assigned to the C-O stretching vibration of ether bonds [20] and confirms the successful incorporation of PEG into the polymer structure.

### 3.3. Gel Permeation Chromatography (GPC)

The characteristics of the new copolymers of PBS-DLS-PEG are presented in Table 2. The molecular weight of the compositions was evaluated using GPC and calculated from ^1^H NMR. It can be seen that in the PBS-DLS copolymer, the calculated amount from NMR is different from the theoretical one. The incorporation of PEG segments was higher as compared to the theoretical calculations, suggesting that in the first step, the consumption of the end groups was higher, resulting in only 4 to 8 wt % incorporation of DLS in copolymers containing PEG of different molecular weights. The values of the number-averaged molecular weight (M_n_) and dispersity index (Đ) (2.6–3.7) are typical for condensation polymers according to GPC/SEC (Table 2) and comparable to the data reported for PBS-DLS [19] and PBS-PEG copolymers [15].

### 3.4. Viscosity and MFI

The results of the intrinsic viscosity (limiting viscosity number, η) and melt viscosity (MFI) for the synthesized polymers containing PEG_1000_ and PEG_6000_ are summarized in Table 3. Overall, the addition of PEG soft segments slightly decreased the limiting viscosity number compared to the PBS-DLS 70-30 copolymer. The addition of PEG_1000_ into PBS-DLS copolymers resulted in a decrease in η from 1.015 to 0.911 dL/g. The incorporation of PEG_6000_ into the polymer structure resulted in higher values of the limiting viscosity number for all copolymers compared to those with PEG_1000_, reaching an η of 0.993 dL/g for the copolymers. These changes are consistent with the literature data, as the limiting viscosity number is associated with the molecular weight of polymers, where a higher molecular weight causes higher viscosity [21].

Regarding the MFI (melt viscosity), the incorporation of PEG_1000_ into the copolymers caused an increase in the MFI values, from 3.16 to 28.01 g/10 min, meaning higher instability during melt processing at a given temperature. Those values increased for PEG_6000_ as a shorter time was needed to measure the MFI at 120 °C. Since a higher molecular weight can cause a greater melt viscosity [22], these results are comparable with the M_n_ of the copolymers obtained from GPC. 

### 3.5. Water Contact Angle (WCA)

**Figure 5:**
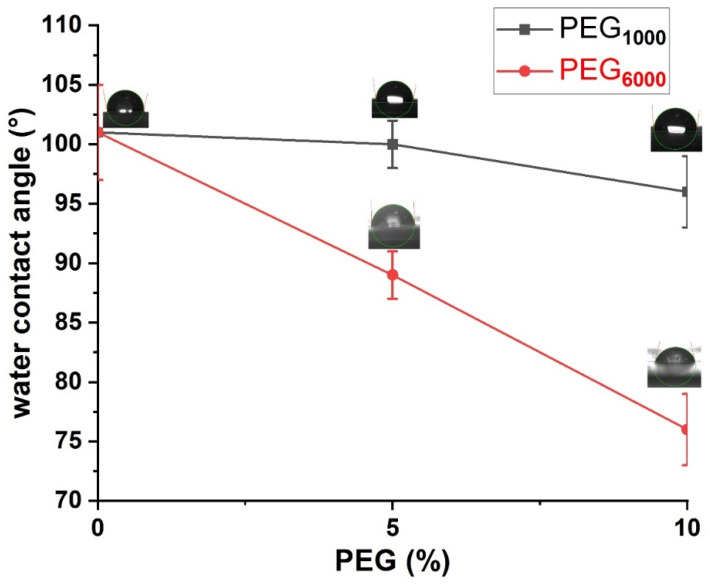
Water contact angle (WCA) change for copolymer series with PEG_1000_ and PEG_6000_ after 60 s.

Polymer wettability is an important factor to take into the consideration, especially for tissue engineering applications for having bifunctional surfaces, as hydrophilicity can increase cell adhesion and proliferation, and this feature can facilitate new tissue growth [23]. Table 4 represents the changes in the contact angles of the drop of water onto the surface of polymer films containing PEG_1000_ and PEG_6000_ after 15, 30 and 60 s. For the PBS-DLS 70-30 copolymer, the presence of 30 wt % of hydrophobic DLS soft segments resulted in an average WCA of 101° after 60 s, thus indicating the highly hydrophobic character of the polymer surface. The incorporation of PEG_1000_ at 5 and 10% reduced the WCA to 100° and 96°, respectively. The effect of PEG_6000_ on the WCA of the copolymers was higher than PEG_1000_ as the WCA of the copolymers was reduced further to 89° and 73°, respectively, by the addition of 5 and 10% of PEG_6000_, indicating the transformation of the surface properties from hydrophobic to hydrophilic. It should be mentioned that the decrease in the WCA was observed in parallel with the increasing content of PEG and increasing molar mass of PEG (similar trend in the increasing intensity of NMR signals was observed). The changes regarding the WCA changes for the copolymers, with the picture of the drop of water onto the surface after 60 s, are also presented in Figure 5. The highest change in the WCA was noticed for samples containing PEG_6000_, thus imparting highly hydrophilic surfaces.

### 3.6. Thermal Analysis

**Figure 6:**
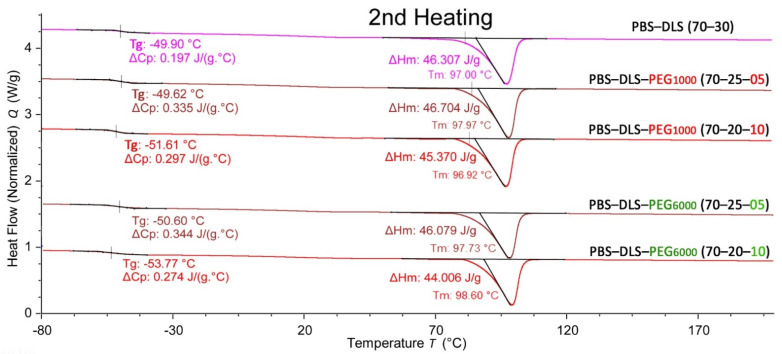
The second heating DSC thermograms for PBS-DLS, PBS-DLS-PEG_1000_ and PBS-DLS-PEG_6000_ of different segmental compositions.

**Figure 7:**
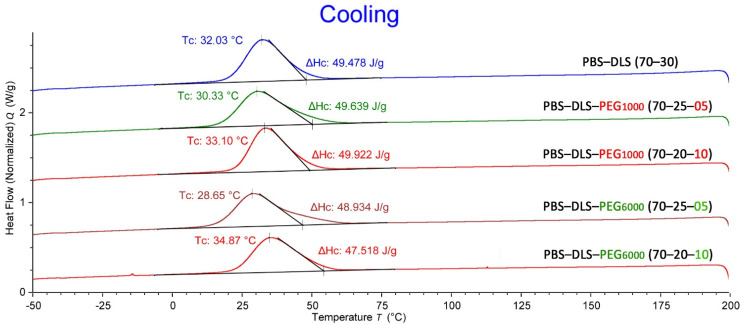
The DSC cooling thermograms for PBS-DLS, PBS-DLS-PEG_1000_ and PBS-DLS-PEG_6000_ of different segmental compositions.

The synthesized copolymers are crystallizable thermoplasts; therefore, the melting temperature was assessed from the second scan to erase the thermal history. The second heating and cooling DSC thermograms for the polymeric series containing PEG_1000_ and PEG_6000_ are shown in Figure 6 and Figure 7, respectively. The values of melting, glass transition temperature and crystallinity are also reported in Table 5 for all materials. From the analysis of the second heating curves of synthesized materials (Figure 6), two distinct transitions can be detected: the low-temperature glass transition (T_g_), ascribed to soft segments, and the high melting temperature (T_m_), characteristic for crystalline hard segments. The appearance of separate T_g_ and T_m_ indicates a microphase separation characteristic for thermoplastic elastomers [24]. The PBS-DLS copolymer revealed the T_g_ at −49.9 °C and the melting peak at 97 °C. Introducing PEG_1000_ and PEG_6000_ into the polymer structure (5 and 10 wt %) slightly shifted the T_g_ to lower values, indicating slightly improved microphase separation. Small changes in the T_m_ were obtained by the addition of PEG_1000_ and PEG_6000_, which is also reflected in the crystallization temperature (T_c_) changes shown in the DSC cooling thermograms (Figure 7). The T_c_ for all copolymers, independently of the amount of PEG_1000_, was detected at 33 °C. 

The observed changes in the thermal properties are also reflected in the calculated degree of crystallinity (X_c_) of the hard segments (Table 5). Generally, the addition of PEG_1000_ and PEG_6000_ did not have a significant effect on the thermal properties of the copolymers, such as T_g_, T_m_, T_c_ and X_c_.

### 3.7. Dynamic Mechanical Properties

**Figure 8:**
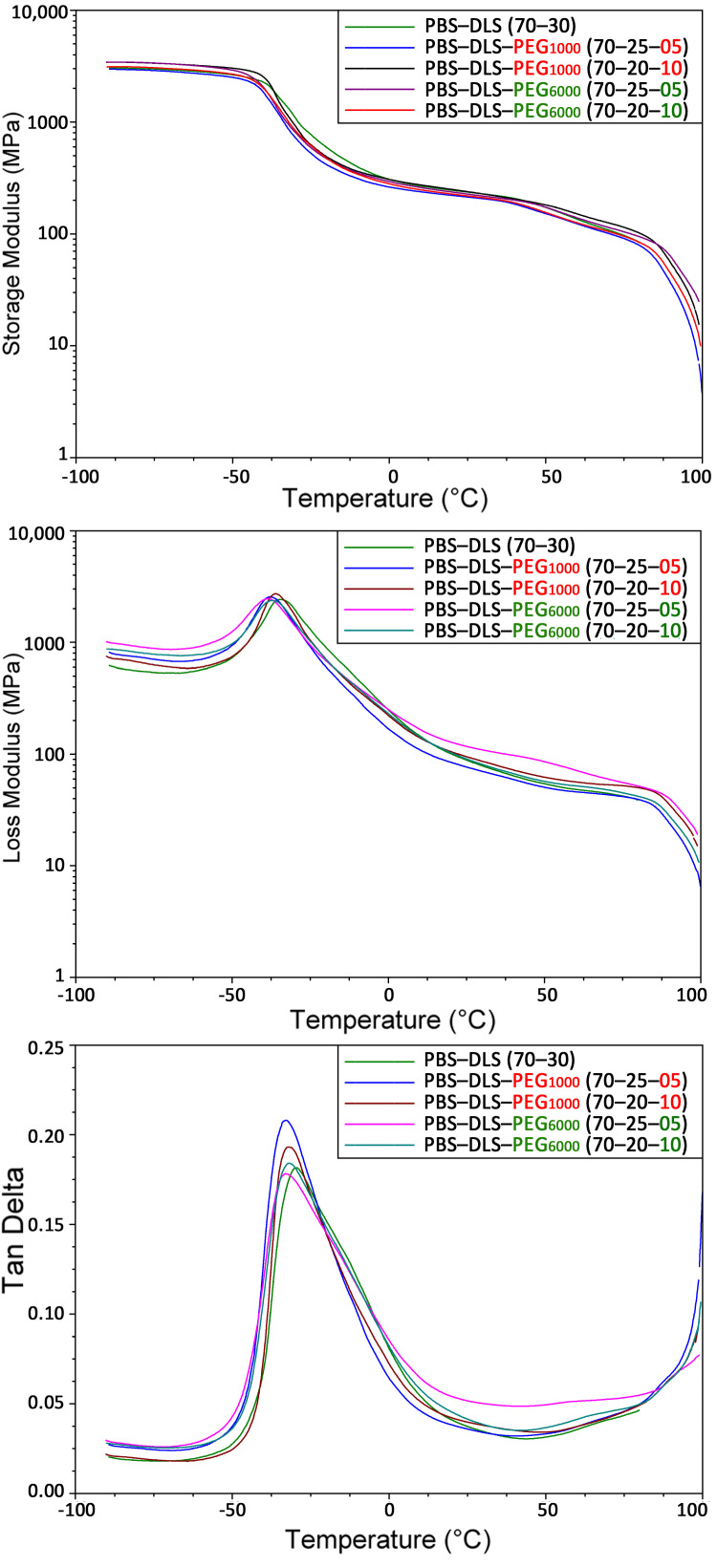
Dynamic thermomechanical analysis (DMTA) of newly synthesized copolymers of PBS-DLS-PEG: storage modulus, G’ (top), loss modulus, G’’ (middle), and tangent of delta, tan δ (bottom).

Dynamic thermomechanical analysis (DMTA) was used to evaluate the effects of different molecular weights of PEG on the relaxation changes in the PBS-DLS-PEG copolymers. The DMTA results of the synthesized PBS-DLS-PEG are presented in Figure 8. The results from the storage modulus in the presence of PEG_1000_ and PEG_6000_ show slight changes compared to the PBS-DLS copolymer. The storage modulus (G’) started shifting down at the T_g_ which is around −45 °C. After the addition of PEG (both PEG_1000_ and PEG_6000_), the values of G’ in the transition region were slightly lower than those for the PBS-DLS copolymer, indicating a lower degree of crystallinity. In the rubbery plateau region, the G’ values for samples with PEG_1000_ and PEG_6000_ ended at a higher temperature compared to the PBS-DLS material, which indicates a higher M_n_. The values of the loss modulus (G’’) curves (measure of the viscous behavior of the copolymers) are also presented in Figure 8. In the presence of PEG_1000_, the maximum values appeared at around −35 °C, with almost no change between the copolymers. However, by the addition and increasing the amount of PEG_6000_, the relaxation maxima shifted to a lower temperature from −35 to −38 °C. The analysis of the damping properties is presented in tan δ curves, shown as the maximum of the T_g_ temperature. The addition of PEG (both PEG_1000_ and PEG_6000_) triggered more intensive damping properties for the copolymers at the subzero region around the T_g_ temperature, and the highest values of tan δ were observed for the copolymers with the highest PEG content (PBS-DLS-PEG 70-20-10). The tan δ curves of the samples also clearly indicate the high homogeneity (sharp peaks) of the amorphous phase. Table 6 shows and compares the values of the maximum G” and the maximum of tan δ ascribed to the T_g_, for both copolymer series with different amounts of PEG_1000_ and PEG_6000_. 

### 3.8. Filament Forming from Polymer Melt

**Figure 9:**
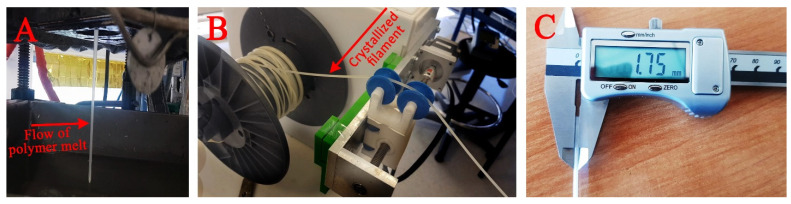
The flow of the polymer melt (PBS-DLS-PEG_6000_ 70-20-10) directly into a water bath (**A**); the rotating collector at the end of the water bath with adjustable turning speed (**B**); and the diameter of the obtained filament after adjusting the speed of the collector (**C**).

The synthesis of new PBS-DLS-PEG copolymers was performed in a stainless-steel reactor (3 l volume), enabling the collection of the polymer melt into the filament. Figure 9 represents the formation of the crystallized filament directly after the reaction (PBS-DLS-PEG_6000_ 70-20-10 is shown as an example). When the reaction was complete, the flowing hot polymeric material was collected directly into a cold water bath. By changing the speed of the collector, the diameter of the filament was adjusted to the standard size of filament suitable for a 3D printer (1.75 mm). The advantages of obtaining the filament directly from the reactor are straightforward as the need for the post-process manufacturing of the filament through extrusion is eliminated. Moreover, a higher-quality and more uniform filament is obtained without the need to use dedicated tools or devices controlling the filament diameter.

## 4. Conclusions

New copolymers containing PBS as a hard segment and DLS and PEG as soft segments were successfully synthesized by two-step polycondensation using a new heterogenous catalyst. The chemical characteristics indicated the incorporation of hydrophilic poly(ethylene glycol) of different molecular weights into the polymer structure. The incorporation of PEG of different molecular weights and amounts resulted in different wettability, thus changing the copolymer characters from hydrophobic to hydrophilic. The results from thermal analysis indicate two distinct transitions—the low T_g_ ascribed to soft segments and the high T_m_ characteristic for crystalline hard segments. The higher molecular weight of PEG caused a higher viscosity. Overall, the thermal properties did not change by the addition of PEG up to 10%. The obtained results indicate the formation of new copolymers with similar thermal properties to the PBS-DLS copolymer, but with sufficiently improved hydrophilicity, which is advantageous for potential tissue engineering applications. Moreover, filament formation directly from the polymer melt makes this new material very promising for fused deposition modeling (FDM).

## Figures and Tables

**Table 1:** A summary of the synthesis conditions and copolymer compositions.

Material	Composition	Transesterification		Polycondensation	
		Time (min)	Temperature (°C)	Time (min)	Temperature (°C)
PBS-DLS	70:30	80	180	140	236
PBS-DLS-PEG_1000_	70:25:5	100	216	140	234
PBS-DLS-PEG_1000_	70:20:10	100	217	140	237
PBS-DLS-PEG_6000_	70:25:5	100	210	140	236
PBS-DLS-PEG_6000_	70:20:10	80	217	140	240

**Table 2:** The molecular weight calculated from ^1^H NMR results and from GPC, the weight and molar percentage ratio between the hard and soft segments.

	Composition (wt %)		*DP_h_*		^1^H NMR	GPC		
Copolymer	Theoretical	Calculated	Theoretical	Calculated	M_n_ (KDa)	M_n_ (KDa)	M_w_ (KDa)	Đ
PBS-DLS	70-30	64-36	8.4	6.53	53.5	49.5	127.5	2.6
PBS-DLS-PEG_1000_	70-25-05	82-08-10	10.09	8.69	20.1	43.3	128	2.8
PBS-DLS-PEG_1000_	70-20-10	64-04-32	12.61	9.52	24.8	32.2	92.4	2.9
PBS-DLS-PEG_6000_	70-25-05	81-06-13	10.09	7.30	60.2	45.6	169.7	3.7
PBS-DLS-PEG_6000_	70-20-10	60-07-33	12.61	9.43	34.1	22.7	69	3.0

**Table 3:** Intrinsic viscosity (limiting viscosity number) and melt flow index (MFI) of the synthesized copolymers with different amounts of PEG_1000_ and PEG_6000_.

Copolymer	Limiting Viscosity Number (dL/g)	MFI (g/10 min)	Time (s)
PBS-DLS (70-30)	1.015	3.16 ± 0.1	30
PBS-DLS-PEG_1000_ (70-25-5)	0.960	13.92 ± 0.8	15
PBS-DLS-PEG_1000_ (70-20-10)	0.911	28.01 ± 1.08	15
PBS-DLS-PEG_6000_ (70-25-5)	1.008	8.66 ± 0.34	30
PBS-DLS-PEG_6000_ (70-20-10)	0.993	136± 1.8	15

**Table 4:** Summary of the water contact angle of the copolymer surface measured at different times.

Copolymer	15 s	30 s	60 s
PBS-DLS (70-30)	104° ± 3°	102° ± 4°	101° ± 4°
PBS-DLS-PEG_1000_ (70-25-5)	102° ± 2°	101° ± 2°	100° ± 2°
PBS-DLS-PEG_1000_ (70-20-10)	98° ± 2°	97° ± 3°	96° ± 3°
PBS-DLS-PEG_6000_ (70-25-5)	94° ± 3°	90° ± 3°	89° ± 2°
PBS-DLS-PEG_6000_ (70-20-10)	76° ± 4°	74° ± 3°	73° ± 3°

**Table 5:** Summary of DSC analysis of newly synthesized PBS-DLS-PEG copolymers with two different molecular weights of PEG_1000_ and PEG_6000_.

Samples	T_g_ (°C)	ΔCp (J/g°C)	T_c_ (°C)	ΔH_c_ (J/g)	T_m_ (°C)	ΔH_m_ (J/g)	Xc _PBS_ (%)
PBS-DLS 70:30	−49.9	0.197	32.0	49.48	97.0	46.31	45.4
PBS-DLS-PEG_1000_70:25:5	−49.6	0.335	30.3	49.64	97.9	46.70	45.8
PBS-DLS-PEG_1000_70:20:10	−51.6	0.297	33.1	49.92	96.9	45.37	44.5
PBS-DLS-PEG_6000_70:25:5	−50.6	0.344	28.7	48.93	97.7	46.08	45.2
PBS-DLS-PEG_6000_70:20:10	−53.8	0.274	34.9	47.52	98.6	44.01	43.1

**T**_g_—glass transition temperature, ΔC_p_—heat capacity, T_c_—crystallization temperature, ΔH_c_—crystallization enthalpy, T_m_—melting temperature, ΔH_m_—melting enthalpy, X_c_—degree of crystallinity of hard segments.

**Table 6:** The comparison of T_g_ values taken at max G’’ and max tan δ.

Copolymers	Max G” (°C)	Max tan δ (°C)
PBS-DLS 70:30	−35.8	−29.8
PBS-DLS-PEG_1000_ 70:25:5	−36.1	−31.8
PBS-DLS-PEG_1000_ 70:20:10	−37.9	−32.8
PBS-DLS-PEG_6000_ 70:25:5	−36.9	−31.7
PBS-DLS-PEG_6000_ 70:20:10	−38.8	−33.3

## Data Availability

The research data are available from the authors upon request.
